# 3D Visualization of the Initial *Yersinia ruckeri* Infection Route in Rainbow Trout (*Oncorhynchus mykiss*) by Optical Projection Tomography

**DOI:** 10.1371/journal.pone.0089672

**Published:** 2014-02-28

**Authors:** Maki Ohtani, Kasper Rømer Villumsen, Helene Kragelund Strøm, Martin Kristian Raida

**Affiliations:** Research Group of Fish Diseases and Immunology, Section of Veterinary Clinical Microbiology, Department of Veterinary Disease Biology, Faculty of Health and Medical Sciences, University of Copenhagen, Frederiksberg, Denmark; State Key Laboratory of Pathogen and Biosecurity, Beijing Institute of Microbiology and Epidemiology, China

## Abstract

Despite the fact that enteric redmouth disease (ERM) in farmed rainbow trout is one of the most devastating disease problems, little is known about the initial route of infection and pathogenicity of the aetiological agent, *Yersinia ruckeri*. In order to determine the initially infected organs, optical projection tomography (OPT), a novel three-dimensional (3D) bio-imaging technique, was applied. OPT not only enables the visualization of *Y. ruckeri* on mucosal surfaces but also the 3D spatial distribution in whole organs, without sectioning. Rainbow trout were infected by bath challenge exposure to 1×10^8^ CFU/ml of *Y. ruckeri* O1 for 1 hour. Three fish were sampled for OPT and immunohistochemistry (IHC) 1, 10 and 30 minutes, 1, 3, 6, 12 and 24 hours, as well as 2, 3, 7 and 21 days after the start of the infection period. *Y. ruckeri* was re-isolated from the blood of infected fish as early as 1 minute post infection. Both OPT and IHC analysis confirmed that the secondary gill lamellae were the only tissues infected at this early time point, indicating that *Y. ruckeri* initially infects gill epithelial cells. The experimentally induced infection caused septicemia, and *Y. ruckeri* was found in all examined organs 7 days post infection including the brain, which correlated with the peak in mortality. To the best of our knowledge this is the first description of *Y. ruckeri* infection in the brain, which is likely to cause encephalitis. This in part could explain the lethality of ERM in rainbow trout. Using OPT scanning it was possible to visualize the initial route of entry, as well as secondary infection routes along with the proliferation and spread of *Y. ruckeri*, ultimately causing significant mortality in the exposed rainbow trout. These results demonstrate that OPT is a state-of-the-art technique capable of visualizing pathogenesis at high resolution.

## Introduction

*Yersinia ruckeri* is a Gram-negative, rod shaped bacterium, causing enteric redmouth disease (ERM) in rainbow trout. The bacterium was initially isolated from diseased rainbow trout in American fresh water aquaculture in the 1950s [1]–[3]. Today it is isolated in many other countries around the world [4]. Various fish species can be infected with *Y. ruckeri* with or without clinical signs, but especially rainbow trout fry are susceptible [5]–[9]. ERM in rainbow trout farming can in part be controlled by vaccination [10]–[14]. Nevertheless, the majority of antibiotics used annually in Danish freshwater aquaculture, is prescribed for rainbow trout infected with *Y. ruckeri* serotype O1 [15]. Despite the devastating economic and animal welfare effect of ERM disease in aquaculture, little is known about the infection route of *Y. ruckeri* as well as the pathogenesis in fish [16].

Some waterborne fish pathogenic bacteria have been shown to adhere to mucus layers and cross the epithelial surfaces covering the gills, skin and gastrointestinal (GI) tract [16], [17]. While healthy skin is covered with scales and mucus, the bacteria, including *Y. ruckeri* O1 biotype 2, entered through injured skin and the lateral line canal [18], [19]. Furthermore, bacteria swallowed with water, surviving passage through the fish stomach enduring low pH and digestive enzymes, are capable of invading the intestinal mucus layer and villi [16], [19], [20]. In fish gills, only two cell layers separate the surrounding water and blood along the pillar capillary network of the secondary lamellae, which enables efficient respiratory gas exchange [21]. However, this feature makes the gills a susceptible tissue for bacterial infection. Zapata and colleagues have shown that *Y. ruckeri* infects through the pavement cells covering the secondary lamellae [22]. Tobback *et al.* also found high quantities of live *Y. ruckeri* in the pavement cells during infection. They further revealed the presence of bacteria within the capillaries and endothelial cells in the lamellae [16]. In addition to this, McIntosh and colleagues demonstrated that *Y. ruckeri* infects the gill epithelia, using an isolated, perfused rainbow trout head setup [23]. Even latex particles coated with *Y. ruckeri* O-antigen and formalin killed *Y. ruckeri* have been shown to be taken up by endocytosis in pavement cells [24].

Recently, Méndez and coworkers performed a sophisticated study of the progression of *Y. ruckeri* infection in rainbow trout, using bioluminescence imaging [25]. Unfortunately the resolution of the IVIS® Imaging System was too low to detect the presence of *Y. ruckeri* until the bacterial cells had multiplied enough to provide a detectable signal 12 hours post bath infection. The IVIS® Imaging System provides a two-dimensional overview of the disease progression in low resolution. However, the technique is currently not applicable for investigating the initial sites of bacterial attachment and early penetration of epithelia cells.

Optical projection tomography (OPT) was developed as a new imaging technique in order to analyze the gene expression and activity with three-dimensional (3D) images [26] and has subsequently seen increasing use in human, mouse, chicken, zebrafish, fruit fly (*Drosophila*) and plant models [27]–[32].

Compared to other methods such as whole mount immunohistochemistry (WIHC) and bioluminescence imaging, the advantage of OPT is its ability to generate a high resolution 3D image from the fluorescent signal of specifically stained cells in the whole organs and at the same time obtain details of the tissue structure. Therefore, we adapted the OPT technology to explore the initial site of *Y. ruckeri* infection, as well as to follow the progression and distribution of *Y. ruckeri* in rainbow trout developing lethal ERM disease. The OPT results were confirmed by conventional IHC, which provides a higher resolution than OPT but is limited to study thin tissue sections in two dimensions only. The objective of the present study was to discover the initial route of infection of *Y. ruckeri* in rainbow trout and to describe the pathogenesis, causing lethal ERM disease.

## Materials and Methods

### Ethics Statement

The study was licensed by the National Animal Experimentation Board (license nr. 2012/561-147) according to the EU Directive EU 86/609. The rainbow trout were treated in accordance with the Animal Experimentation Act of Denmark, which is in accordance with the Council of Europe Convention ETS 123.

### Fish

Rainbow trout were hatched and reared under pathogen-free indoor conditions at the Aquabaltic Hatchery (Nexø, Denmark), and transferred to the experimental fish facility at the University of Copenhagen (Frederiksberg, Denmark) prior to experimental challenge. The pathogen-free bacterial status of the rainbow trout fry was controlled upon arrival at the university by standard bacteriology methods. The average weight of the fish used in this study was 5.4±2.5 g. The fish were kept at 18°C water temperature in 128 liter aquaria with internal biofilters (Eheim biofilters, Germany) and air supply ensuring saturated aeration. Commercial feed pellets (BioMar A/S, Denmark) were hand fed to the fish once per day.

### *Yersinia ruckeri*

*Y. ruckeri* serotype O1 biotype 1 strain 392 isolated from diseased rainbow trout [33] was cultured in Luria Bertani broth for 48 hours at 23°C with shaking (100 rpm). For the challenge experiment, cells were pelleted by centrifugation (4000 *g* for 10 min.) and after removal of the growth medium, the bacteria were re-suspended in 18°C tap water. The number of colony forming unit (CFU) per ml water used for bath infection was quantified by triplicate plating of a ten-fold dilution series of the bacteria on blood agar plates (State Serum Institute, Denmark), as described earlier [14], [34].

### Challenge experiment

The challenged rainbow trout fry (n = 112) were bath infected in 10 L water containing 1×10^8^ CFU/ml *Y. ruckeri* O1 biotype 1 for 1 hour, with constant aeration of the challenge aquarium. Subsequently, the fry were transferred back to their 128 L aquarium containing clean, aerated tap water. The control group of un-infected fish was kept under identical conditions in clean water. The fish were examined a minimum of three times a day, in order to record and remove moribund individuals during the 21 days of challenge. Rainbow trout exhibiting a loss of equilibrium or showing isolated behavior, dark coloration of the skin and loss of appetite were regarded as moribund and were euthanized. Rainbow trout that survived the 21 days challenge experiment were all euthanized by an overdose of MS-222 (100 mg/L) (Sigma-Aldrich, Denmark).

### Sampling

At 1, 3, 6, 12 and 24 hours post infection (hpi) as well as 2, 3, 7 and 21 days post infection (dpi), 6 fish were euthanized by an overdose of MS-222 and sampled. Blood was sampled from *vena caudalis* of all 6 fish using heparinized syringes. The 6 fish euthanized per sampling time point were divided into two groups of three fish each, and fixed either in 10% formalin for immunohistochemistry or 4% paraformaldehyde (PFA) for OPT.

### Additional infection experiment for sampling at early time points

In the initial challenge experiment described above the earliest sampling time point was 1 hpi. Since *Y. ruckeri* was found to be present in the blood of all the sampled trout already at this stage, an additional short time (1 h.) infection experiment was conducted two weeks after the onset of the main challenge experiment, to sample infected fish at even earlier time points. In this infection experiment, a total of 36 rainbow trout from the same cohort of fry were challenged for 1 h in 10 L of *Y. ruckeri* suspended in aerated water to a concentration of 1×10^8^ CFU/ml as in the first challenge. Four fish were euthanized and blood was sampled from each fish 0, 1, 5, 10, 20, 30, 40, 50 and 60 mpi. The four fish sampled 1, 10, 30 and 60 minutes post infection (mpi) were after blood samplings fixed for OPT and IHC. Three of the fish were fixed in 4% PFA for OPT and 1 fish per time point were fixed in 10% formalin for IHC.

### Quantifying *Y. ruckeri* in the blood

To determine the CFU of *Y. ruckeri* in the blood of infected fish, 10 µl of blood from four fish per time point was diluted in tenfold dilution steps in phosphate buffered saline (PBS), and 10 µl of each dilution were plated on blood agar plates in triplicate. The CFU/ml was counted two to three days later, as previously described [35].

### Purification of anti-*Y. ruckeri* rabbit IgG

To avoid nonspecific background staining in the OPT scanning, rabbit anti-*Y. ruckeri* IgG was purified from anti-*Y. ruckeri* rabbit serum [36], using a HiTrap Protein G column (GE Healthcare, UK) according to the manufacturer's instruction. Briefly, 1 ml of rabbit serum was diluted with 9 ml of binding buffer (1.5 M glycine-NaOH, 3 M NaCl, pH 8.9) and filtered through a 0.45 µm filter. Subsequently, the filtrated serum was applied to the HiTrap Protein G column and washed with 10 ml of binding buffer. Finally, the rabbit IgG was eluted with elution buffer (0.1 M sodium citrate, pH 6.0, 5.0 and 4.0) and each fraction (1 ml/fraction) was immediately neutralized with 1 M Tris-HCl (pH 9.0).

### Optical projection tomography

Tissue samples were fixated in freshly prepared 4% PFA in PBS (pH 7.4) for 3 h at 4°C. After washing in PBS for 30 min, tissues were dehydrated in increasing methanol concentration (33%, 66% and 100%) for 15 min per step. Next, tissues were incubated in methanol: dimethyl sulfoxide (DMSO):30% H_2_O_2_ (2∶1∶3) at room temperature overnight to quench autofluorescence. After two consecutive washes in 100% methanol for 30 min, tissues were kept at −80°C after which 4−5 freeze/thaw cycles were performed, allowing samples to thaw for at least 1 hour at room temperature before returning them to −80°C again, to ensure that antigens in the deeper parts of the tissue were rendered accessible through cell lysis. Tissues were then rehydrated in increasing TBST (2.5 mM Tris-HCl, 4.5 mM NaCl, 0.01% Triton X-100, pH 7.4) concentration (33%, 66% and 100%) in methanol for 15 min per step. Tissues were incubated in blocking solution (10% goat serum and 0.01% NaN_3_ in TBST) for 24 h at room temperature, and then incubated with 5,000 fold diluted, purified anti-*Y. ruckeri* rabbit IgG in blocking solution containing 5% DMSO for 48 h at room temperature. Tissues were washed extensively with TBST overnight and incubated with 2,000 fold diluted secondary antibodies Alexa Fluor® 594 conjugated goat anti-rabbit IgG (A11037, Life technologies) for 48 h. Tissues were washed with TBST overnight, and then embedded in 1% ultrapure low melting agarose (Life technologies). The embedded agarose blocks were trimmed to an optimum size (1 cm×1 cm×1.7 cm) and dehydrated in 100% methanol over-night. Finally, the sample was cleared in BABB solution (benzyl alcohol: benzyl benzoate, 1∶2) overnight, in order to become transparent. The BABB cleared specimen was attached to a metal disc using glue for the final scanning procedure. For the projection of samples, Bioptonic 3001 M OPT scanner (Bioptonic Inc., UK) was used, and a series of 800 images were captured per 360° rotation. Bioptonic 3001 M OPT scanner has two light sources visible light (transmission light) and UV light with three filters (GFP1, GFP and Cy3), and we used UV light with GFP1 and Cy3 filters for detection of autofluorescence and AlexaFluor® 594 for detecting the presence of *Y. ruckeri*, respectively.

The captured images were reconstructed with NRecon software (Bruker microCT, Belgium) and then arranged as 3D image with Bioptonic Viewer software (Bruker microCT). Overlaying of the captured images was performed by Adobe Photoshop CS6, and any possible artifacts were removed manually. 3D movies were made using QuickTime Pro.

### Immunohistochemistry

Tissue samples were fixed in 10% formalin overnight at 4°C, and then transferred to 70% ethanol until processed. The immunohistochemical procedure has been described by Chettri and colleagues [36]. In brief, after sectioning on a microtome (Leica, Germany), sections (4 µm) were placed on SuperFrost slides (Thermo scientific, USA) and dried at 40°C overnight. The rabbit anti-*Y. ruckeri* serum was diluted 100,000 fold for IHC. The peroxidase labeled polymer conjugated to goat anti-rabbit immunoglobulins in EnVision®+ System-HRP (DakoCytomation, Denmark) was used as secondary antibody. Color was developed with AEC substrate (Thermo scientific).

The Na^+^/K^+^ ATPase (NKA) has been used as a marker molecule to identify chloride cells in fish [37], [38]. Therefore, an avian NKA-specific mouse monoclonal antibody was used for double fluorescent immunohistochemistry to identify the chloride cells in gill epithelia. The dehydrated sections were blocked with 4% goat serum in PBS at room temperature for 20 min, and then covered with 100,000 fold diluted anti-*Y. ruckeri* polyclonal antibody overnight at 4°C. After wash with PBS for 5 min, the sections were covered with 500 fold diluted Alexa Fluor® 594 conjugated goat anti-rabbit IgG (A11037, Life techologies) at room temperature for 1 hour. After wash with PBS, the sections were covered with 100 fold diluted supernatant containing the anti-avian NKA alpha subunit mouse monoclonal antibody (clone a5, Developmental Studies Hybridoma Bank, University of Iowa, USA) at 4°C overnight. After wash with PBS, the sections were covered with 500 fold diluted Alexa Fluor® 488 conjugated goat anti-mouse IgG antibody (A11001, Life techologies) at room temperature for 1 hour. After wash with PBS, the sections were mounted and observed by fluorescent microscopy.

### Statistical analysis of results

All statistical analyses were conducted using GraphPad PRISM software (GraphPad software Inc., USA), and p-values <0.05 were considered statistically significant. Analysis for differences in mortality between infected and un-infected groups of rainbow trout was performed using a Kaplan-Meier log-rank test. In order to test for differences in CFU/ml blood between different time points, a Kruskal-Wallis test with Dunn's multiple comparison post test was conducted.

## Results

### Yersinia ruckeri O1 bath challenge

**Figure 1:**
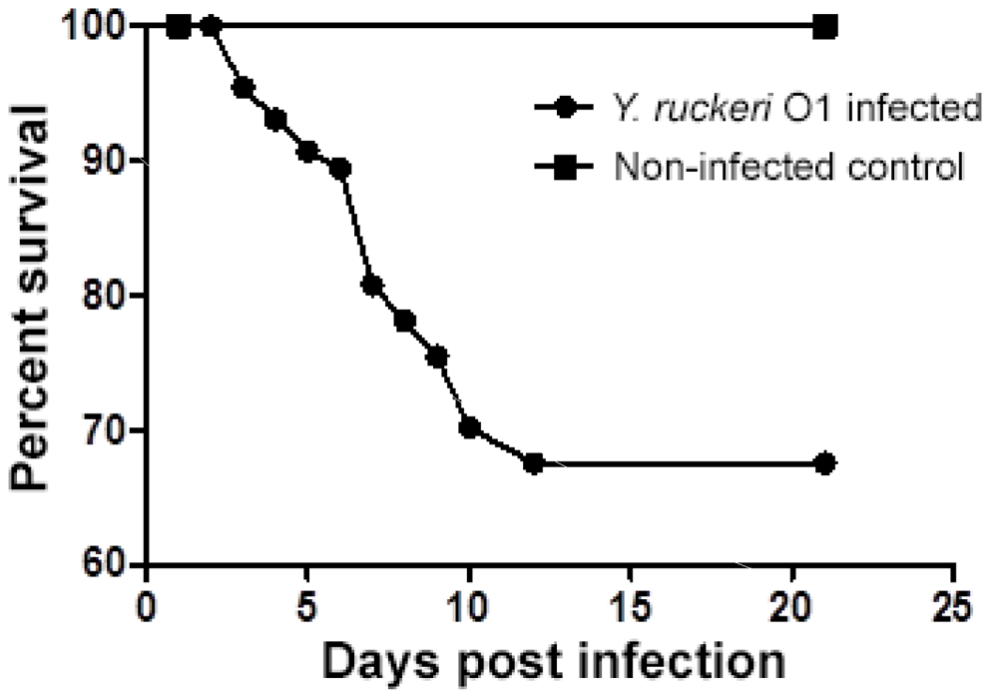
Mortality of rainbow trout during experimental bath infection with *Y. ruckeri*. The fish (n = 112) were exposed to *Y. ruckeri* O1 (1×10^8^ CFU/ml) in one aquarium for 1 hour and then moved to an aquarium with clean water for 21 days of observation. There was significantly cumulative mortality in the infected group compared with the un-infected group by use of Kaplan-Meier survival analysis (*p*<0.01).

During the 21 days of the main challenge experiment, 33% mortality was observed in the infected group, whereas no mortality was observed in the uninfected control group. Mortality in the infected group was observed between day 3 and 12 post infection, with the highest daily mortality rate observed at 7 dpi (Fig. 1). The cumulative mortality of the *Y. ruckeri* infected group was significantly higher than that of the control group (*p*<0.01).

### *Y. ruckeri* count in blood post infection

**Figure 2:**
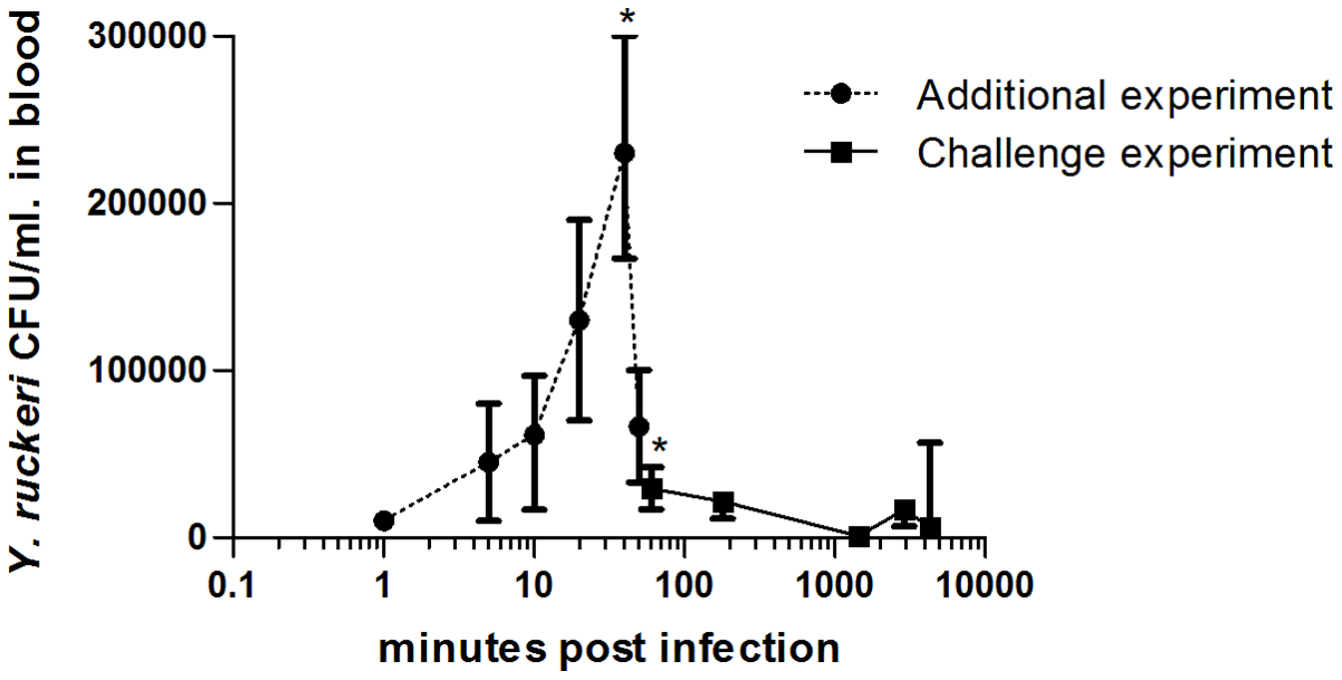
Detection of *Y. ruckeri* in blood of infected trout. Blood was sampled from uninfected control and *Y. ruckeri* infected fish during the challenge experiments (n = 4). Blood samples were taken from 1 hpi until 3 days post infection during the initial challenge (solid line). Since all infected trout had *Y. ruckeri* in the blood already at 1 hpi, an additional experiment was conducted and blood samples were taken between 1 mpiand 1 hpi (dashed line). *Y. ruckeri* were re-isolated from all blood samples obtained from all infected fish at all-time points and are given as medians with ranges in the figure. Kruskal-Wallis test with Dunn's multiple comparison post-test was used to test for difference in the quantity of *Y. ruckeri* in the blood in infected relative to uninfected rainbow trout. The infected group had significantly increased numbers of *Y. ruckeri* in the blood 40 mpi and 1 hpi *p* = 0.0279 and *p* = 0.0076, respectively. * = *p*<0.05.

In order to confirm systemic *Y. ruckeri* infection in the rainbow trout, throughout the experimental challenge, blood samples were collected from four fish at 1, 10, 20, 40 50 and 60 mpi in the additional infection experiment, and 1, 24, 48 and 72 hpi in the main challenge experiment. *Y. ruckeri* was re-isolated from all blood samples taken during this study, but we were unable to count the CFU in some highly infected samples, due to confluent bacterial growth on blood agar plates. In the infected group, *Y. ruckeri* was detected at 1 mpi and the median value increased until 40 mpi where the CFU/ml peaked (2.3×10^5^, range 1.3×10^5^ CFU/ml), after which they declined (2.9×10^4^, range 2.5×10^4^ CFU/ml) at 60 mpi (Fig. 2). The median value of CFU per ml blood decreased (1.0×10^3^, range 2.3×10^3^ CFU/ml) at 24 hpi, but then rose again at 48 hpi (1.5 ×10^4^, range 1.2×10^4^ CFU/ml). There was a significant amount of *Y. ruckeri* in the blood at 40 mpi and 60 mpi with *p* = 0.0279 and *p* = 0.0076, respectively, relatively to the negative pre-infection samples.

### 3D scanning of entire organs from rainbow trout

**Figure 3:**
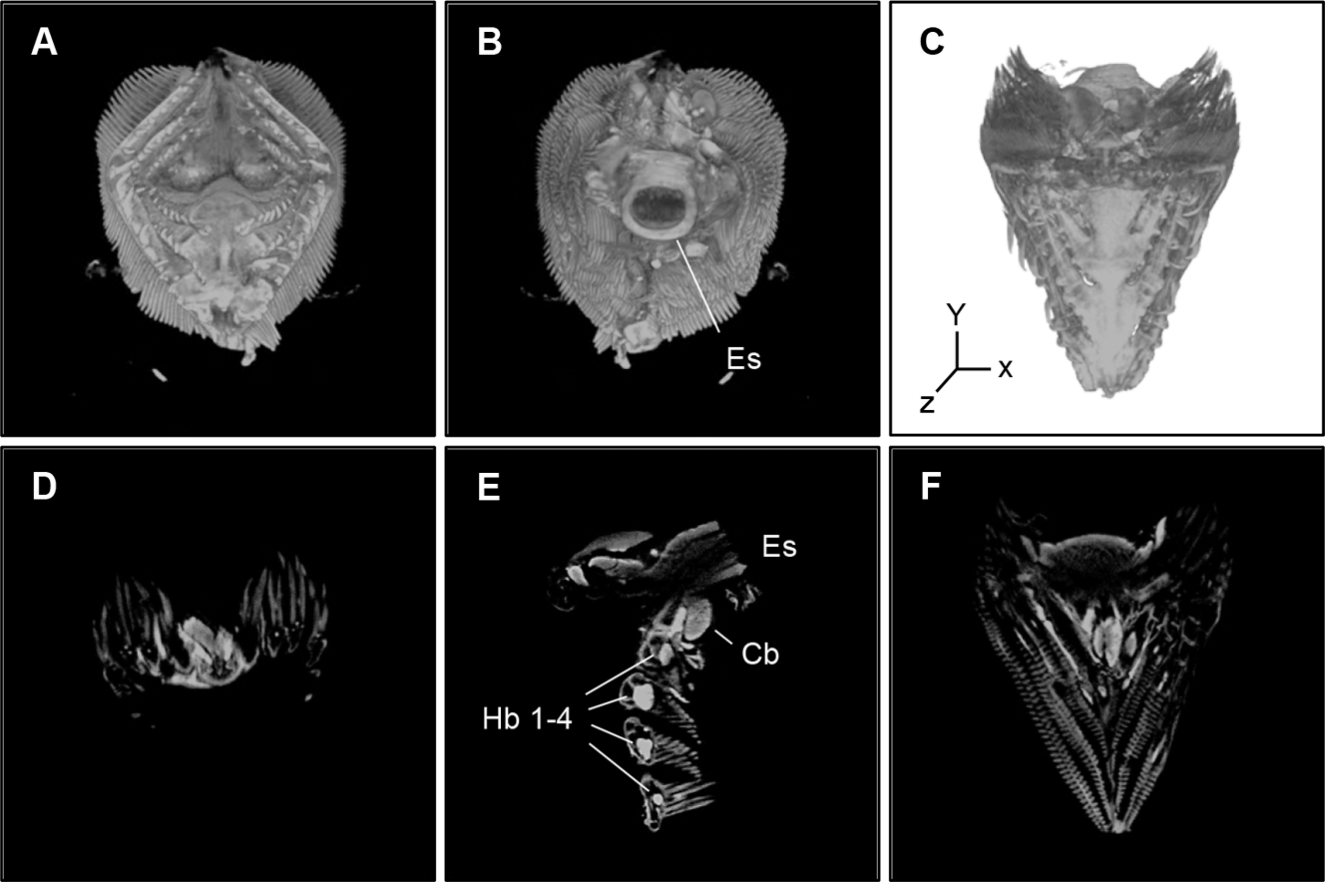
Anatomy of the gills of rainbow trout based on detection of autofluorescence. 3D organization and images of vertical sections of the gills from a healthy non-infected rainbow trout. The image are taken from the following angles: (A) proximally, (B) distally, (C) overlaid images of autofluorescent showing the anatomy and TXR red channel (negative control showing no binding of *Y. ruckeri* specific antibodies). (D-F) 2D sections generated from a 3D model of C along (D) coronal axis, (E) sagital axis, (F) transveral axis. Esophagus, Es; hypobranchial, Hb; ceratobrancial, Cb.

We successfully visualized the 3D organization of gills, GI tract, liver, spleen, heart, brain, skin and kidney by OPT. Figure 3 showing the anatomy of the gills based on detection of autofluorescence in a non-infected rainbow trout (Fig. 3). As showed in Fig. S1, 3D organization and transversal or sagittal sections of organs were produced, enabling exploration of the internal organs (see Supporting Information, Movie S1).

### Detection of *Y. ruckeri* in gills, skin and kidney

**Figure 4:**
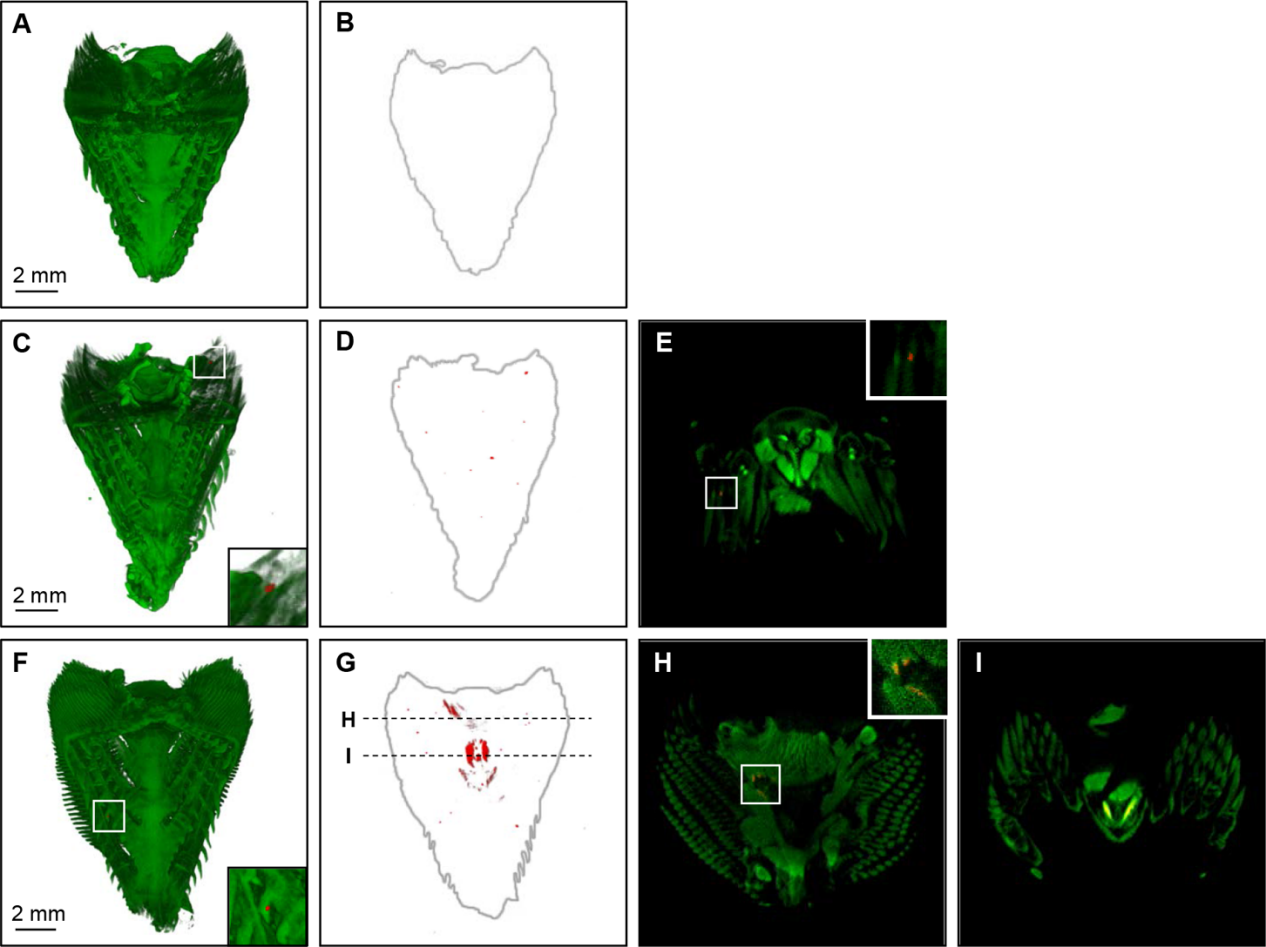
Detection of *Y. ruckeri* in the gills of infected rainbow trout by OPT. (A, C and F) Red spots showing specifically stained *Y. ruckeri* fluorescent and green is autofluorescent showing the gill morphology. B, D and G show the overview of the *Y. ruckeri* positive signals detected in the sample (grey outline).E, H and I show the *Y. ruckeri* specific staining in vertical sliced sections. A and B are the gills from an uninfected rainbow trout (negative control) sampled pre infection, showing no *Y. ruckeri* specific staining (red). (C−E) Gills sampled at 1 mpi demonstating that *Y. ruckeri* is attached to the gill lamellae and it is detected inside the lamellae (E). (F−I) Show the progression of *Y. ruckeri* infection over time, detected in the gills and esophagus at 7 dpi.

The organs dissected from *Y. ruckeri* infected fish were stained with anti-*Y. ruckeri* rabbit IgG and Alexa Fluor® 594 conjugated goat anti-rabbit IgG antibody. The *Y. ruckeri* specific positive signal appeared in the epithelia covering the gill lamellae at 1 mpi (Fig. 4C), and the number of spots had increased at 10 mpi (Fig. S2A). The horizontal section at 1 mpi shows that *Y. ruckeri* was associated with the gill filament (Fig. 4E). *Y. ruckeri* infected cells in the gill filament were still seen 7 dpi, where *Y. ruckeri* had proliferated further (Fig. 4G, and see Supporting Information, Movie S2 and S3 ). These proliferations of *Y. ruckeri* were localized at the surface of the esophagus and the connective tissue near the gills as well (Fig. 4H, 4I). These *Y. ruckeri* specific infected spots were detected at every time point post infection by OPT (Fig. S2). No positive signals were observed in samples from un-infected control fish (Fig. 3 A-F, 4B, and see Supporting Information, Movie S3), strongly indicating that the fluorescent positive signal was *Y. ruckeri* specific.

**Figure 5:**
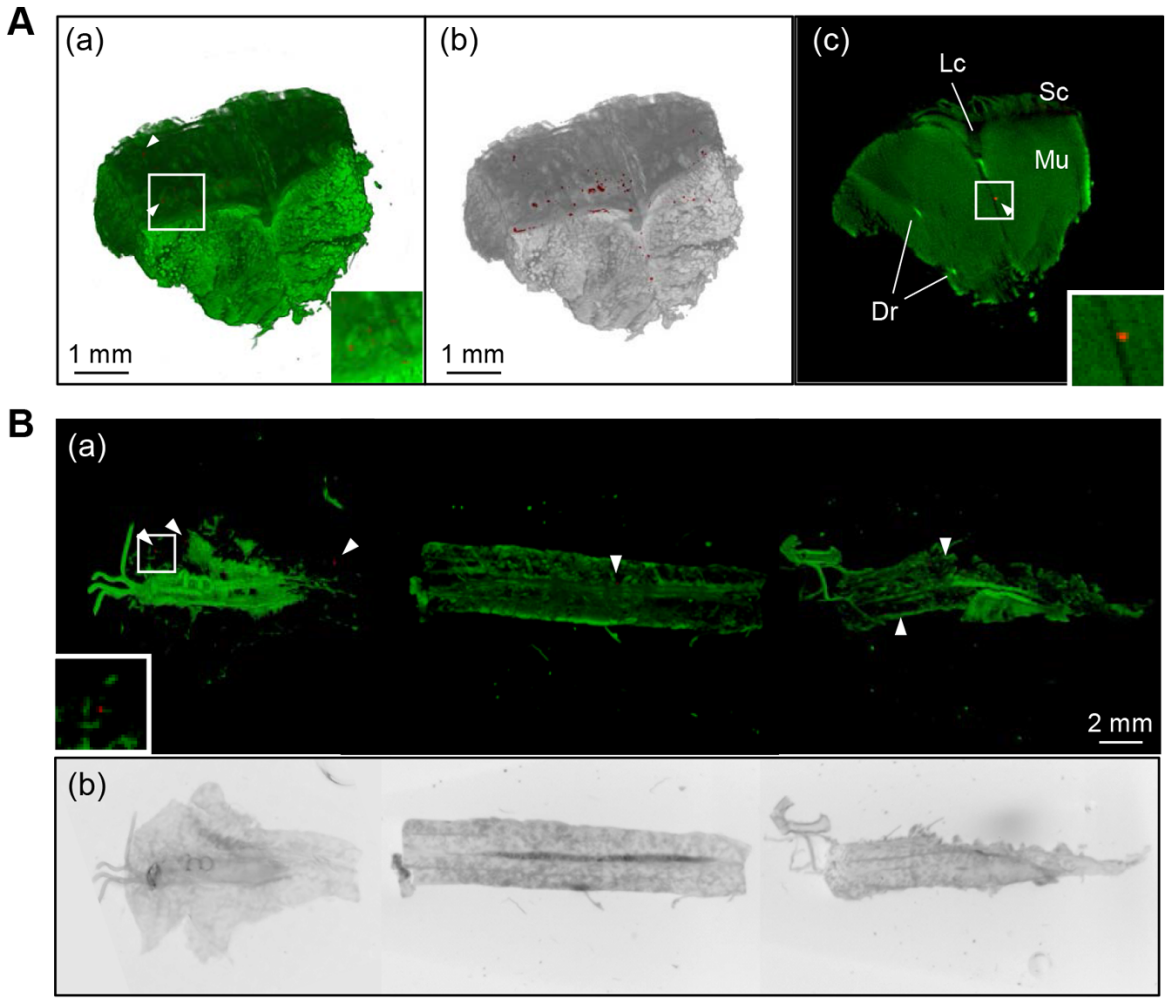
Detection of *Y. ruckeri* in skin and kidney. (A) 3D spatial organization of skin and underlying muscles surrounding the lateral line canal (sampled 10 mpi). (a) Shows the 3D structure based on autofluorescence (green) and only *Y. ruckeri* present on the outer surfaces of the 3D sample is displayed. Picture (b) shows (a) as a flattened 2D image, resulting in an overlaid figure enabling the display of every *Y. ruckeri* positive signal throughout the entire 3D model in a single 2D section. (c) The vertical section of the skin including *Y. ruckeri* positive signal from the deeper part of the connective tissue (arrowhead). Lateral line canal (Lc); scale, Sc; skeletal muscle, Mu; dorsal rib bone, Dr. (B) The 3D spatial organization of whole kidney sampled at 3 dpi. In order to scan the whole kidney, it was cut into three pieces. (a) The overlay of autofluorescence and *Y. ruckeri* specific staining generate an image which shows that *Y. ruckeri* positive signals could be detected throughout the kidney. Green (a) and gray (b) colors display the anatomy of the whole kidney in 3D and flattened 2D, respectively.

*Y. ruckeri* was also detected on the epidermis and in the lateral line canal in the skin 10 mpi (Fig. 5A), as well as in the head and trunk kidney 3 dpi (Fig. 5B). *Y. ruckeri* was not detected in sufficient amounts to give a positive signal by OPT scanning in the GI tract, liver, spleen, heart and brain (data not shown).

### Detection of *Y. ruckeri* in rainbow trout organs by IHC

**Figure 6:**
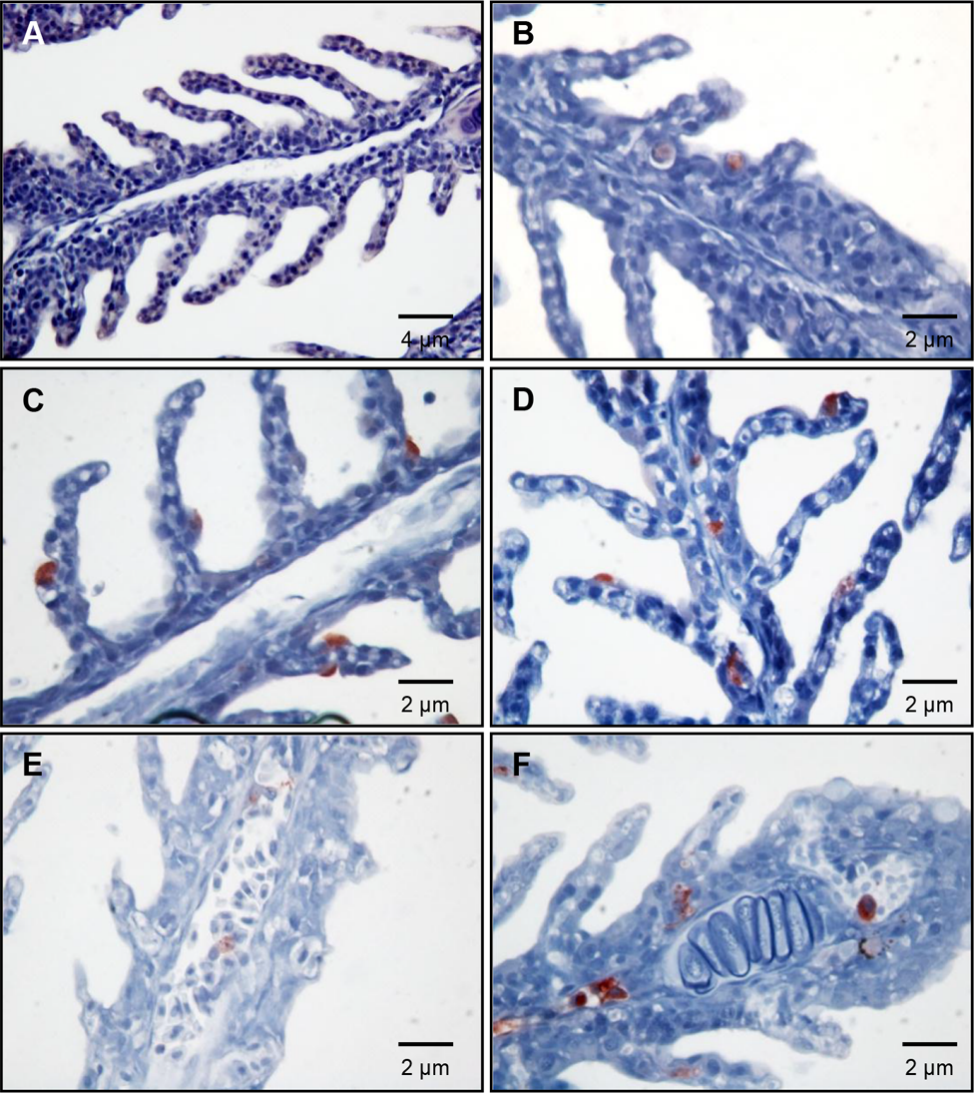
Immunohistochemistry of gill lamellae from un-infected and *Y. ruckeri* infected rainbow trout. The sections are from (A) uninfected control, (B) 1 mpi, (C) 10 mpi, (D) 6 hpi, (E) 48 hpi and (F) 7 dpi and were stained with rabbit anti-*Y. ruckeri* polyclonal antibody and HRP conjugated goat anti-rabbit IgG antibody. The nuclei were counterstained with hematoxylin. *Y. ruckeri* was detected inside the gill lamellae already at 1 mpi and the blood capillaries in the gill lamellae at 7 dpi, indicating systemic infection.

**Figure 7:**
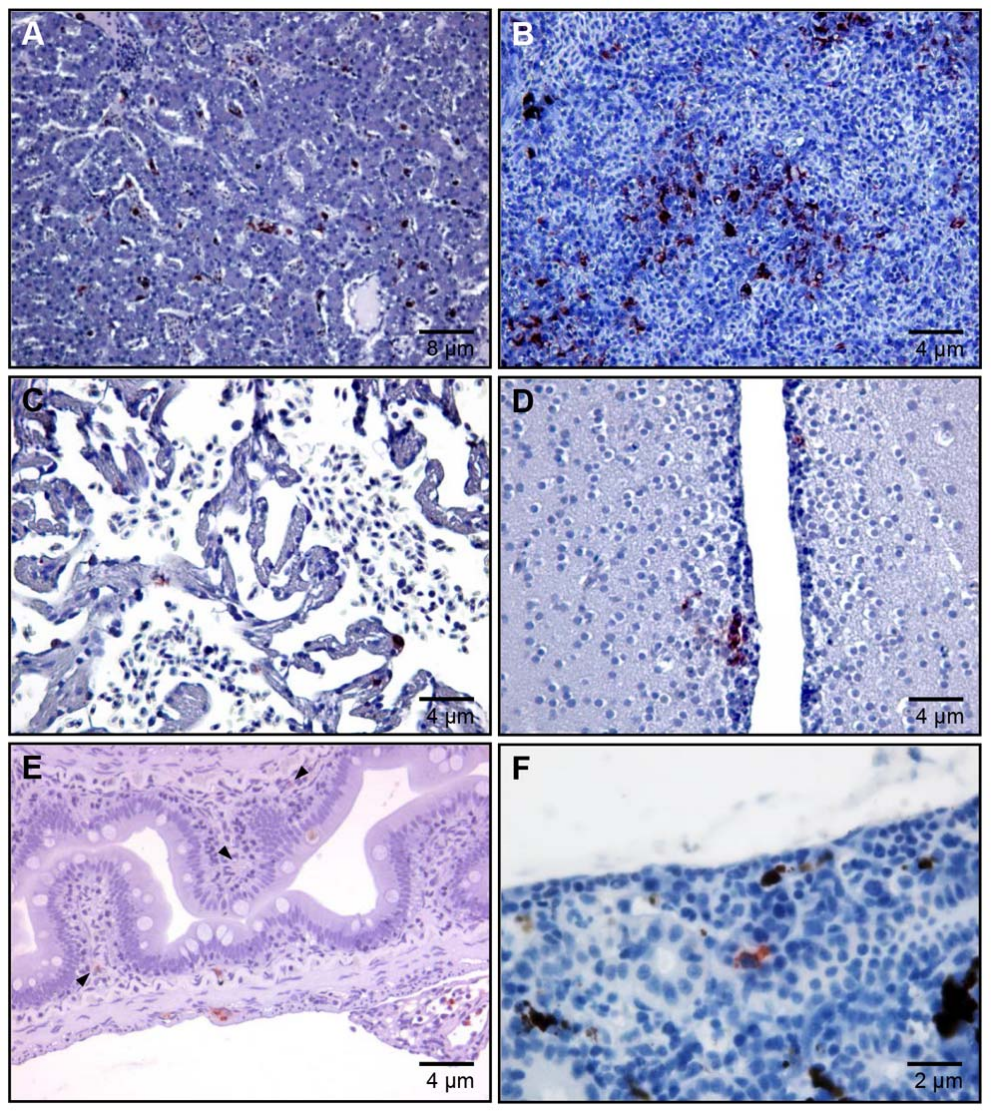
Immunohistochemistry of internal organs from *Y. ruckeri* infected rainbow trout. The sections are from (A) liver, (B) spleen, (C) heart, (D) brain (optic tectum) and (E) intestine sampled 7 dpi, and (F) trunk kidney sampled 3 dpi, and stained as described in Fig. 6.

Organs collected from infected fish at each sampling time point, were used for conventional IHC to confirm the *Y. ruckeri* infection detected by OPT, in order to achieve even higher resolution. *Y. ruckeri* was found in gill epithelia at 1 and 10 mpi, 1, 3 and 6 hpi (Fig. 6B, 6C). Furthermore, *Y. ruckeri* was present not only in the epithelial cells but also in the endothelial cells of blood capillaries at 6 hpi (Fig. 6D). Monocytes containing *Y. ruckeri* in their cytosol appeared in the blood capillaries at 48 hpi, and severe septicemia was seen 7 dpi (Fig. 6F). *Y. ruckeri* infected cells were found neither in gills from control fish (Fig. 6A), nor challenged fish sampled at 21 dpi (data not shown). Large quantities of *Y. ruckeri* were seen in the lumen of the intestine, adhering to mucus and invading villi from the lumen at 30 mpi (Fig. S3D). A few *Y. ruckeri* infected cells were found in the trunk kidney at 3 dpi, however it was difficult to see the infected cells at the 7 dpi because of increased infiltration of melanomacrophages (Fig. 7F and data not shown). Furthermore *Y. ruckeri* was detected in the liver, spleen, brain, heart and intestine at 7 dpi (Fig. 7), but not observed in liver, spleen, brain or heart at other time points. The resolution of IHC is higher than for OPT. Hence, by using IHC we were able to detect single *Y. ruckeri* bacteria in tissue sections, which was not achievable by OPT.

### Distinction of *Y. ruckeri* infected gill epithelial cells

**Figure 8:**
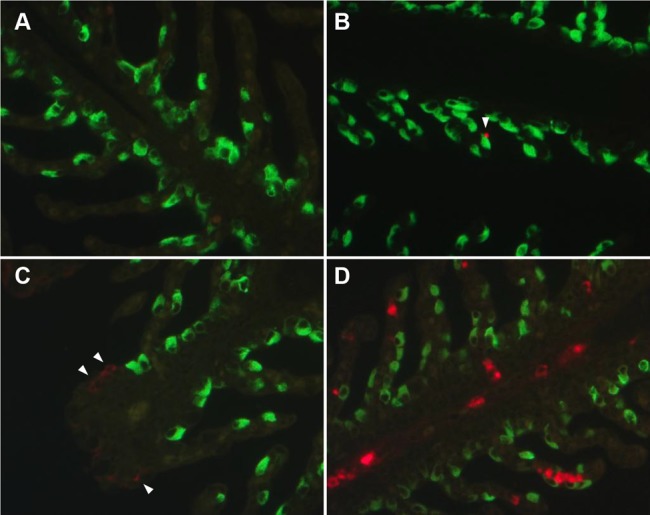
Double fluorescent immunohistochemistry of the gill lamellae from un-infected and *Y. ruckeri* infected rainbow trout. The *Y. ruckeri* ^+^ and NKA^+^ cells are visualized in red and green, respectively. Sections of gill lamellae from (A) un-infected control, (B) 10 mpi, (C) 6 hpi and (D) 7 dpi are stained, as described in materials and methods. The arrowheads indicate the *Y. ruckeri* ^+^ cells. No *Y. ruckeri* (red) were detected in the chloride cells (green).

The cytosol of the NKA positive (NKA^+^) chloride cells were stained specifically (green color), and found near the base of secondary gill lamellae, where the chloride cells are expected to be located (Fig. 8A). A single *Y. ruckeri* cell was detected near to, but not co-localized with a NKA^+^ cell 10 mpi (Fig. 8B). At six hpi, numerous *Y. ruckeri* infected cells appeared on the tip of the secondary gill lamellae, while still not co-localized with NKA^+^ cells (Fig. 8C). Although the number of infected cells was highly increased at 7 dpi, *Y. ruckeri* and NKA^+^ double positive cells were absent (Fig. 8D). The lack of co-localization between *Y. ruckeri* bacteria and NKA^+^ cells was observed at all examined time points as well (data not shown).

## Discussion

In order to describe and follow the initial infection route of *Y. ruckeri*, is it important to use an experimental infection model that effectively mimics a natural infection, where the bacteria is left to cross the immediate physical barriers of the host immune defense to establish an infection, as would be the case outside of an experimental setup during a real breakout of ERM. At the same time, the model must be controllable and allow for accurate sampling at the designated time points. Bath infection of rainbow trout with *Y. ruckeri* O1 biotype 1 strain 392 [33] is a well-established bath infection model fulfilling these criteria [14], [34]. To investigate why *Y. ruckeri* causes lethal ERM disease in rainbow trout, it is essential to choose a virulent *Y. ruckeri* wild-type strain that is able to cause significant mortality among experimentally infected rainbow trout. Tobback and colleagues have shown that a large fraction of the *Y. ruckeri* isolates obtained from disease outbreaks in rainbow trout farms are avirulent in experimental bath infections. They speculate that this is related to the less stressful condition found under laboratory conditions compared to fish farms. The avirulent *Y. ruckeri* isolates are able to infect rainbow trout, but are unable to survive within the host for longer periods of time, and therefore do not result in ERM [39].

The initial phases of *Y. ruckeri* infection of mucosal tissues including gills, skin and GI tract have been studied using various methods. However, these methods have been technically limited, because they are based upon very small tissue sections (IHC), or low resolution (bioluminescent imaging) [25]. For example, whole mount *in situ* hybridization (WISH) and WIHC has been conducted on zebrafish [40], [41]. These methods are limited to analysis depth of 1 mm into the tissue. To trace the progression of bacterial pathogens in infected fish, constructs utilizing green fluorescent protein (GFP) tagged *Y. ruckeri*, *Vibrio anguillarum* or *Edwardsiella tarda*, as well as luminescence genes including firefly luciferase genes, or the *lux* operon from *Photorhabdus luminescens* introduced into Koi herpes virus and *Y. ruckeri* respectively, have been developed for detection with fluorescent microscopy, fluorescence-activated cell sorting (FACS) and bioluminescent detecting equipment [25], . Common for these methods is the difficulty to see the spatial distribution of the pathogens in the examined organs. It was further demonstrated that insertion of the GFP gene into *Y. ruckeri,* reduces the virulence [42]. In order to obtain the spatial 3D overview of the distribution of *Y. ruckeri* during initial infection of mucosal tissues, the OPT method was adapted in the present study.

**Figure 9:**
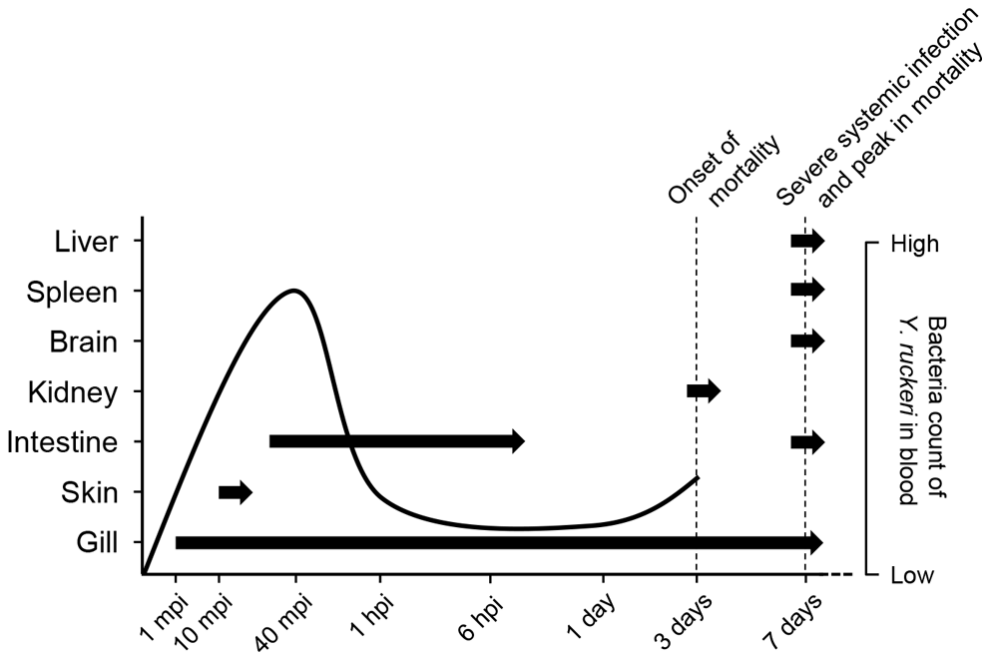
Summarizing the progression of the *Y. ruckeri* infection in blood and organs. Arrows indicate the presence of *Y. ruckeri* in the organ at the time point, where bacterial cells were observed by OPT, and/or IHC. The solid line indicates the change of bacteria counts in blood obtained from this study.

In the current study, *Y. ruckeri* was re-isolated from blood 1 mpi. At this early time point post infection, only gill pavement cells were infected indicating that *Y. ruckeri* found in the blood has entered through the gill epithelial cells. Based on these results, we suggest that *Y. ruckeri* initially infects the gills and that the bacterial infection immediately spreads further to the blood circulation system, and thereby to other internal organs, which are thus secondarily infected at later time points (Fig. 9).

The epithelia covering the secondary gill lamellae consist of an outer layer of chloride- and pavement cells [46]. Combining the OPT and IHC results from our study, *Y. ruckeri* was revealed intracellularly in the epithelial cells 1 mpi. In addition to this, it was discovered that the chloride cells were left un-infected by *Y. ruckeri*, indirectly suggesting that the pavement cells, the other of the two cell types covering the secondary lamella, is the initial target for *Y. ruckeri* in rainbow trout. Zapata *et al.* have previously demonstrated the presence of *Y. ruckeri* in the cytoplasm of pavement cell in rainbow trout by transmission electron microscopy [22]. *Y. ruckeri* infection of gill epithelial cells has been shown using both *Y. ruckeri* O1 biotype 1 and 2 [16], [19] and Tobback and colleagues have previously suggested that *Y. ruckeri* spread rapidly from the epithelial cells to the blood capillary in the gill lamella [16], which is in agreement with the results presented here. Although the exact details of the molecular mechanisms for *Y. ruckeri* infection in gill epithelia remain unknown, we suggest that the pavement cells are the primary route of infection. With invasion blocking assay it has been demonstrated earlier that treatment of *Y. ruckeri* with colchicine and cytochalasis-D decreases the ability of several strains to invade rainbow trout cell cultures, indicating that microtubules and microfilaments are involved in successful invasion [39]. In an elegant adherence assay, it was shown that *Y. ruckeri* bind twice as efficient to gill mucus, than gut mucus of rainbow trout [39]. During infection with a virulent *Y. ruckeri* O1 (strain 5), twice as many bacteria were found in the gill tissue compared to gut tissue, stressing the importance of the gills an essential route of infection [16], [19].

The OPT results strongly indicated that *Y. ruckeri* adheres to the skin surface and penetrate the deeper cell layers in the lateral line canal after 10 mpi, which has also been shown by Khimmakthong *et al.*, who observed presence of live *Y. ruckeri* in the lateral line canal and basal layer of the skin epidermis after 15 mpi [19]. These results indicate that infection from skin and lateral line represents a secondary infection route relative to the initial, rapid infection of the gills and blood observed in this study (Fig. 2, 4 and 6). While Khimmakthong *et al.* described that live *Y. ruckeri* was detected in esophagus, stomach and posterior intestine after 1 mpi, no information concerning the presence of bacteria in the blood was reported [19].

Tobback *et al.* has shown low numbers of *Y. ruckeri* in the intestinal lumen at early time points post infection [16]. In the present work, the earliest findings of *Y. ruckeri* in the intestine were in villi 30 mpi. Hence, it does not seem to be the initial tissue to be infected, although it is infected later, in part because the bacteria was already present in high quantities in the blood at this time point. On the other hand, large quantities of *Y. ruckeri* were found in the lumen of the intestine between 30 mpi and 6 hpi (Fig. S3) and a *Y. ruckeri* infection of the intestine were demonstrated as could be expected, since *Y. ruckeri* is an enterobacterium. The difference in pathogenesis between studies by Tobback and the data presented here is likely due to the use of two different *Y. ruckeri* O1 biotype 1 strains, whereas Khimmakthong and co-workers in their experiment [16], [19] used a biotype 2 characterized by a lack of flagella, which may influence the early pathogenesis.

Interestingly, the numbers of *Y. ruckeri* in the blood peaked at 40 mpi and then started to decline even though the rainbow trout were exposed to the *Y. ruckeri* bacteria suspension for the duration of the 1 hour bath challenge. The decrease in CFU/ml of *Y. ruckeri* observed at 1 hpi (Fig. 2) indicates a fast clearance of *Y. ruckeri* from the blood, probably due to phagocytosis of the bacteria, a bactericidal effect of innate humoral factors, or because the bacteria leave the bloodstream over the course of the infection. Despite this apparent clearing of *Y. ruckeri* from the bloodstream at early time points, a significant number of the rainbow trout developed a lethal ERM disease. The numbers of *Y. ruckeri* in the blood started to increase again 2−3 days post infection, correlating with the onset of mortality. Identical infection patterns, showing initial increase followed by a decrease and then a final increase in the amount of *Y. ruckeri* again, have previously been described in gills, intestine, liver, spleen and head kidney in *Y. ruckeri* infected rainbow trout [16], indicating a systemic distribution of *Y. ruckeri* in the organs during the infection, probably due to septicemia.

Phagocytosis of *Y. ruckeri* by phagocytic cells isolated from peripheral blood leukocytes and kidney have been demonstrated [42], [47], however, a number of *Y. ruckeri* could survive in phagocytes since *Y. ruckeri* has demonstrated resistance to intracellular killing [48]. Ryckaert and colleagues also showed that *Y. ruckeri* survived inside kidney macrophages for at least 17 days post infection [48]. In the present study, monocytes containing *Y. ruckeri* were observed in the capillaries. Regarding innate immune factors, two types of lysozymes isolated from rainbow trout kidney have shown bactericidal activities against *Y. ruckeri* [49], and there is no evidence that *Y. ruckeri* is able to resist lysozyme activity.

The cumulative mortality of this study was 33%, confirming that strain 392 is highly virulent. Tobback and colleagues revealed that some avirulent *Y. ruckeri* strains showed similar adhesion and invasion ability but low replication within the host compared with virulent strains [19], [38].

Haig *et al.* have demonstrated that mainly serum resistant *Y. ruckeri* isolates are pathogenic for rainbow trout [50]. Serum resistance therefore appears to be a very important virulence factor for primarily extracellular bacteria such as *Y. ruckeri*. The *Y. ruckeri* strain 392 used in the present infection experiment has been shown to be resistant towards rainbow trout plasma [33]. In line with these facts, Davies also suggested that serum resistance adds to the pathogenesis of *Y. ruckeri* infection [51]. The human pathogenic members of the genus *Yersinia*, *Y. enterocolitica* and *Y. pestis*, have a conserved *ail* gene which encodes an outer membrane protein that confers resistance to complement-dependent killing [52], [53], but a similar sequence was not found in *Y. ruckeri* [54].

Severe *Y. ruckeri* infections were observed in liver, spleen, heart and brain at 7 dpi, coinciding with the peak mortality in the infected group. To the best of our knowledge this is the first time that *Y. ruckeri* infection have been detected in the brain (Fig. 7). We speculate that an infection of the brain partly could explain why *Y. ruckeri* O1 biotype 1 (strain 392) is so virulent for rainbow trout. Moribund trout were displaying erratic, spinning swimming patterns, possibly indicating pathological neurological dysfunction caused by the presence of *Y. ruckeri* in the brain. Similar observations have been reported from the histopathology of *Streptococcus iniae*, *Lactococcus garvieae* and *Edwardiera ictaluri* infections that are known to cause encephalitis [55]–[57], which makes neurological dysfunction a plausible explanation for the change in behavior observed in this study.

We were unable to find *Y. ruckeri* positive cells in the kidney 7 dpi, which is most likely because of the increased amount of melanomacrophages in head- and trunk kidney, in which we cannot see the bacteria. Tobback and colleagues described that many dead or surviving fish showed increased level of melanomacrophages in kidney [16]. Melanomacrophages are known to take up antigens [17] and therefore it is likely that they are able to present antigens to lymphocytes in order to activate the adaptive immune system. Rainbow trout surviving an infection with *Y. ruckeri* develop immunity against re-infection due to development of adaptive immunity [34].

To summarize the present study, Fig. 9 was made to present an overview of where *Y. ruckeri* is found during infection. The initial adherence and entry route of *Y. ruckeri* from the water is proposed to be via the secondary lamellae of the gills, and secondary infection routes through the lateral line canal in the skin, and the intestine. The maximal bacteria count in blood was found at 40 mpi. At this time point post exposure, the gills, skin and intestine were likewise infected with *Y. ruckeri*. Vast quantities of bacteria were present in the intestinal lumen at 30 mpi and persisted for at least 6 hours. Proliferation of *Y. ruckeri* was evident from 3 dpi, correlating well with both the observation of clinical signs of ERM, as well as the onset of mortality. At 7 dpi a severe systemic infection was apparent, and mortality peaked.

Gills were the first organs found to be infected. Based on our findings, we suggest that *Y. ruckeri* found in blood 1 mpi may have penetrated via the pavement cells in the gill lamellae and from there spread further to the pillar capillaries. Once *Y. ruckeri* has reached the blood stream it spreads throughout the host. The spatial global distribution of *Y. ruckeri* in whole organs was successfully visualized by OPT and also confirmed by conventional IHC. OPT scanning can successfully be used to study other host-pathogen interactions. The technique offers a great 3D overview of a relative large sample and positive staining can be validated with even higher resolution by IHC, as these two methods supplement each other very well.

## Supporting Information

Figure S1 **Images showing 3D organization and transversal or sagittal sections showing the internal anatomy of various organs from healthy un-infected rainbow trout.** 3D anatomy of the gastrointestinal tract (A, B), liver (C, D), spleen (E, F), heart (F, G) and brain (H, I). Stomach, St; pyloric caeca, Pc; intestine, In; bulbus arteriosus, Ba; atrium, At; ventricle, Ve; cerebellum, Ce; optic tetum, OT; telencephalon, T; olfactory bulb, OB; medulla dolongate, Md.
(TIF)

Figure S2 **Detection of** ***Y. ruckeri*** **inside the gill lamellae by use of OPT.** *Y. ruckeri* (red spot) were detected in gill lamellae sampled at (A) 10 mpi, (B) 1 hpi, (C) 3 hpi, (D) 6 hpi, (E) 12 hpi, (F) 24 hpi, (G) 48 hpi and (H) 3 dpi. The gray line shows the outline of the scanned gills.
(TIF)

Figure S3 **Immunohistochemistry of rainbow trout intestine.** (A) The intestine from un-infected control, (B) 1 mpi, (C) 10 mpi, (D) 30 mpi, (E) 6 hpi and (F) 7 dpi (high magnification of Fig. 7E). The sections are stained with rabbit anti-*Y. ruckeri* polyclonal antibody and HRP conjugated anti-rabbit IgG. The nuclei were counter stained with hematoxylin.
(TIF)

Movie S1 **Image sequence of X and Y axis sections of the heart from a un-infected rainbow trout.** The reconstruction of the heart is based on autofluorescence signal (green).
(MOV)

Movie S2 **3D visualization of** ***Y. ruckeri*** **in gill lamellae 7 dpi.** Isosurface reconstruction of a *Y. ruckeri* infected rainbow trout gill lamellae stained with anti-*Y. ruckeri*. The reconstruction of the gill is based on autofluorescence signal (green) whereas *Y. ruckeri* are visualized in red.
(MOV)

Movie S3 **3D visualization of un-infected gill lamellae.** Isosurface reconstruction of un-infected rainbow trout gill lamellae stained with anti-*Y. ruckeri* antibody. The reconstruction of the gill is based on autofluorescence signal (green), whereas no *Y. ruckeri* specific signal (red) was observed (negative control).
(MOV)
