# Supplementary figures and images for: 3D Visualization of the Initial Yersinia ruckeri Infection Route in Rainbow Trout (Oncorhynchus mykiss) by Optical Projection Tomography
Source: PLoS One. 2014 Feb 28;9(2):e89672. doi: 10.1371/journal.pone.0089672 (PMC3938485; doi:10.1371/journal.pone.0089672)

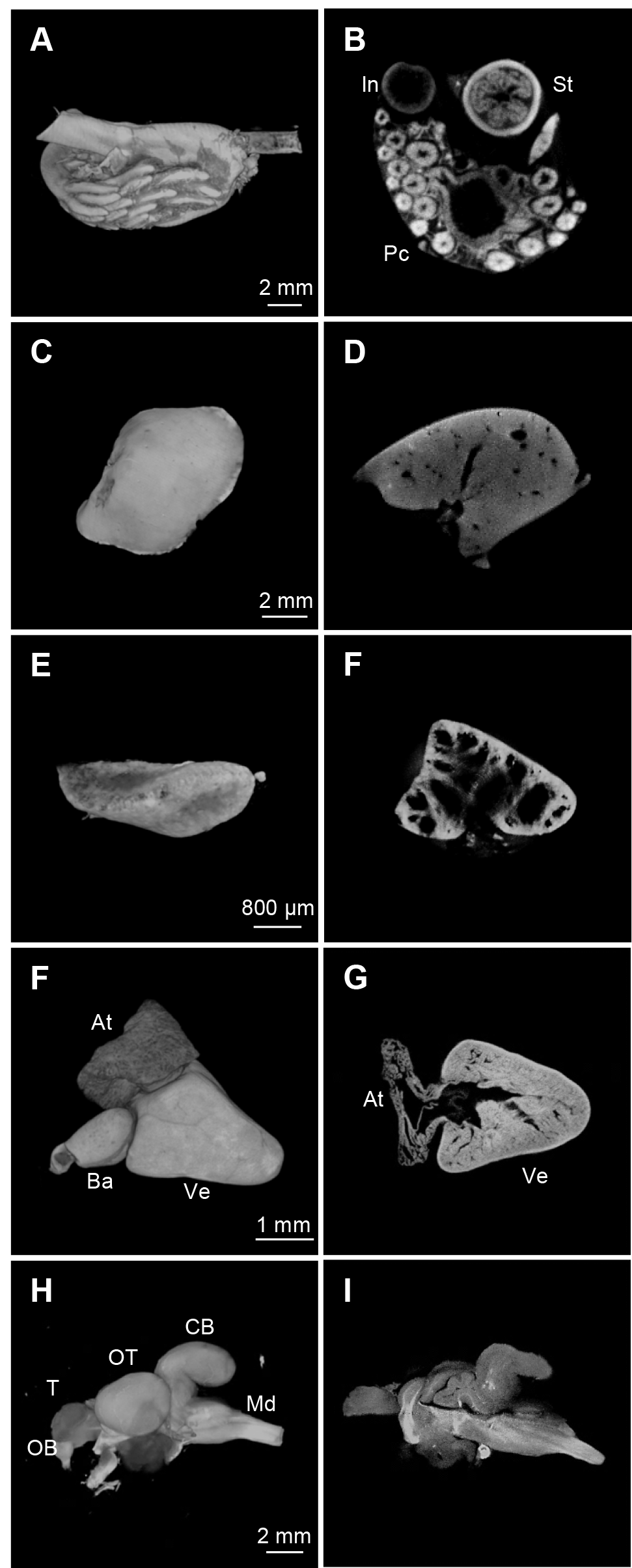

Supplement: Figure S1 — Images showing 3D organization and transversal or sagittal sections showing the internal anatomy of various organs from healthy un-infected rainbow trout. 3D anatomy of the gastrointestinal tract (A, B), liver (C, D), spleen (E, F), heart (F, G) and brain (H, I). Stomach, St; pyloric caeca, Pc; intestine, In; bulbus arteriosus, Ba; atrium, At; ventricle, Ve; cerebellum, Ce; optic tetum, OT; telencephalon, T; olfactory bulb, OB; medulla dolongate, Md. (TIF) [file pone.0089672.s001.tif]

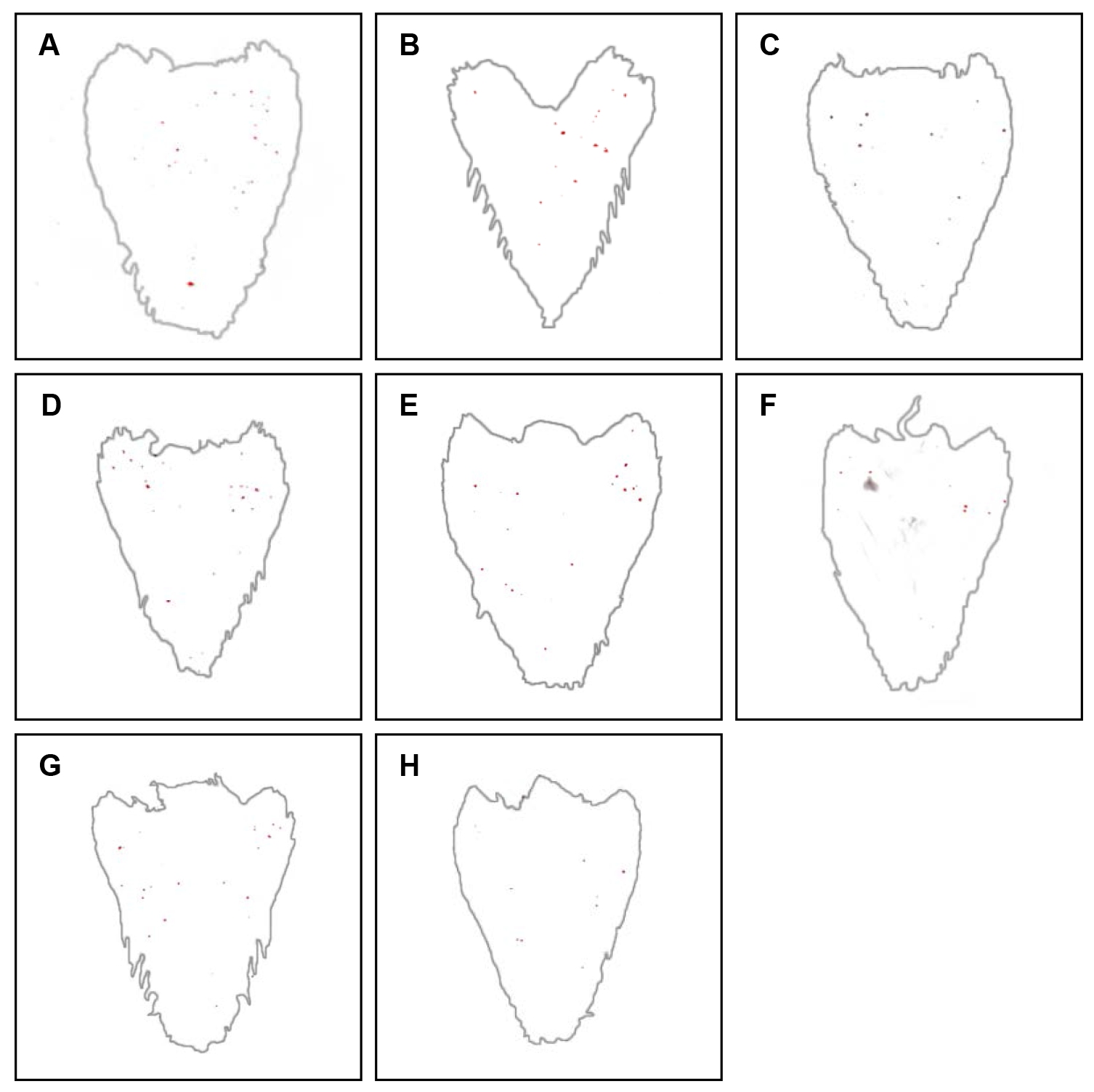

Supplement: Figure S2 — Detection of Y. ruckeri inside the gill lamellae by use of OPT. Y. ruckeri (red spot) were detected in gill lamellae sampled at (A) 10 mpi, (B) 1 hpi, (C) 3 hpi, (D) 6 hpi, (E) 12 hpi, (F) 24 hpi, (G) 48 hpi and (H) 3 dpi. The gray line shows the outline of the scanned gills. (TIF) [file pone.0089672.s002.tif]

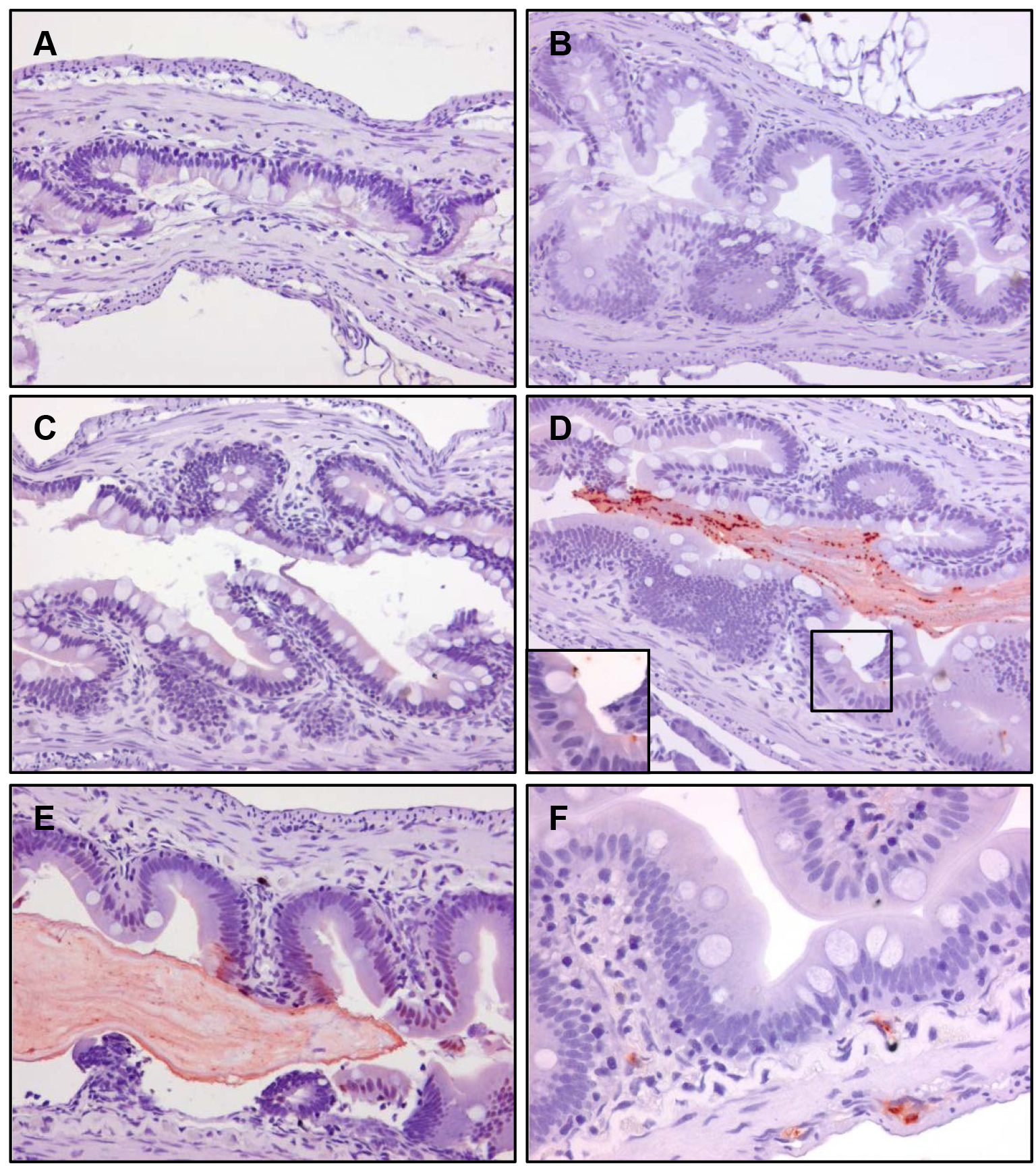

Supplement: Figure S3 — Immunohistochemistry of rainbow trout intestine. (A) The intestine from un-infected control, (B) 1 mpi, (C) 10 mpi, (D) 30 mpi, (E) 6 hpi and (F) 7 dpi (high magnification of Fig. 7E). The sections are stained with rabbit anti-Y. ruckeri polyclonal antibody and HRP conjugated anti-rabbit IgG. The nuclei were counter stained with hematoxylin. (TIF) [file pone.0089672.s003.tif]
